# Survival of Lassa Virus in Blood and Tissue Culture Media and in a Small Particle Aerosol

**DOI:** 10.3390/pathogens9090680

**Published:** 2020-08-21

**Authors:** Sophie J Smither, Lin S Eastaugh, James S Findlay, Lyn M O’Brien, Mark S Lever

**Affiliations:** Chemical Biological and Radiological Division, Defence Science and Technology Laboratory (Dstl), Porton Down, Wiltshire SP4 0JQ, UK; lseastaugh@dstl.gov.uk (L.S.E.); jsfindlay@dstl.gov.uk (J.S.F.); lmobrien@dstl.gov.uk (L.M.O.); mslever@dstl.gov.uk (M.S.L.)

**Keywords:** Lassa virus, survival, aerosol, blood, tissue culture media, stability

## Abstract

Knowledge of the survival and stability of a pathogen is important for understanding its risk, reducing its transmission, and establishing control measures. Lassa virus is endemic in West Africa, causes severe disease, and is an emerging pathogen of concern. Our study examined the survival of Lassa virus in blood and tissue culture media at two different temperatures. The stability of Lassa virus held within a small particle aerosol was also measured. In liquids, Lassa virus was found to decay more quickly at 30 °C compared to room temperature. Sealed samples protected from environmental desiccation were more stable than samples open to the environment. In a small particle aerosol, the decay rate of Lassa virus was determined at 2.69% per minute. This information can contribute to risk assessments and inform mitigation strategies in the event of an outbreak of Lassa virus.

## 1. Introduction

Viruses frequently cause outbreaks of severe disease in humans. These outbreaks can be geographically limited or can quickly spread to infect an entire region or spread globally. There are a range of factors that facilitate the ease by which viruses can spread through a population. One such factor is the stability of the virus on surfaces or in an aerosol. Understanding virus survival is important for decision making to identify the risk from these pathogens and potential mitigation strategies. 

Lassa virus, a member of the Arenaviridae family, causes Lassa (haemorrhagic) fever and is responsible for approximately 5000 deaths a year. The disease, endemic in West Africa, is spread by rats; the main route of transmission is direct contact with rat excreta or items contaminated with rat faeces or urine [1,2]. Nosocomial transmission can occur but person-to-person transmission is rare [1,3]. Lassa virus is classified as a Hazard Group 4 virus (and a US Select Agent) and must be handled under maximum biosafety containment (BSL-4). Lassa virus is present on the WHO’s list of emerging pathogens likely to cause severe outbreaks and for which few or no medical countermeasures exist [4]. Lassa fever normally has a low case fatality rate, but 2018 saw one of the worst ever outbreaks of Lassa fever in Nigeria with a case fatality rate of 25% in confirmed cases. In Nigeria the 2019 case fatality rate was 22% and as of June 2020 was 14.8% [5]. 

Although the main route of transmission is via contact with rodent excreta, there have been instances of nosocomial spread during outbreaks [1,3,6,7]. This may be through contact with bodily fluids, contaminated surfaces, or through the generation of aerosols, which may occur during certain hospital or laboratory procedures [8]. Investigations of Lassa virus survival help inform risk assessments and guide control measures. We investigated the survival of Lassa virus in tissue culture media and blood, and within a small particle aerosol to add to the existing information about the hazard of Lassa virus.

## 2. Results

### 2.1. Survival of Lassa Virus in Blood and Tissue Culture Media

**Figure 1:**
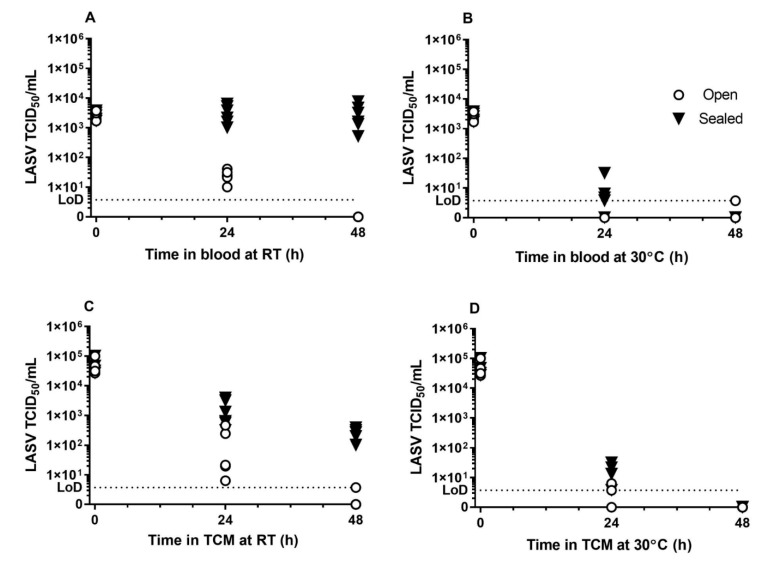
Titre of Lassa virus in blood or tissue culture media over time. The persistence of Lassa virus in blood (top row, (**A**,**B**)) or tissue culture media, TCM, (bottom row, (**C**,**D**)) was measured over two days. Samples were stored at room temperature (RT, 19–22 °C, left, (**A**,**C**)) or 30 °C (right, (**B**,**D**)), open to the external environment (white circles) or sealed (black triangles). Experiments were performed on two separate occasions and the individual titres from three replicates per experiment are shown (*n* = 6 per condition). The limit of detection (LoD) of the TCID_50_ assays is shown as a dotted line on each graph.

To determine virus stability on surfaces, Lassa virus was incubated either in Dulbecco’s Modified Eagle tissue culture medium (TCM) or rat’s blood (for a more representative clinical matrix) over a 48 h time period within polypropylene tubes. The virus was incubated at 30 °C or room temperature (RT: 19–22 °C); at both temperatures, virus was either left exposed (open tube) or sealed to the environment. Small volumes (20 µL) were used to mimic real-life scenarios in clinical settings, such as droplets generated by coughing or blood splatter. Droplets held at 30 °C or in open tubes at room temperature all dried. Virus in TCM and blood within sealed tubes at room temperature remained as droplets over 48 h. The titres of viable Lassa virus within blood or TCM maintained at 30 °C open, or sealed to the environment, decreased rapidly over 24 h. No virus or only viral titres at the limit of detection could be detected after 48 h (Figure 1B,D). The titres of Lassa virus, in blood or TCM held at room temperature (RT) but open to the environment, also decreased over 24 h and no virus (blood) or no or very low titres of viable virus (TCM) could be detected at 48 h (Figure 1A,C). The only conditions where titres of Lassa virus could be detected after 48 h were in sealed tubes held at room temperature in either matrix (Figure 1A,C).

Survival data were fitted to the one-phase exponential decay model so the decay constant could be determined (Table 1). Decay constants varied from 0.149 to 0.411. A decay constant for blood at room temperature, sealed to the environment, could not be determined as no decay was observed over 48 h. A decay constant for blood at 30 °C, open to the environment, could not be determined as no viable virus was recovered at 24 h. 

### 2.2. Survival of Lassa Virus in a Small Particle Aerosol

**Figure 2:**
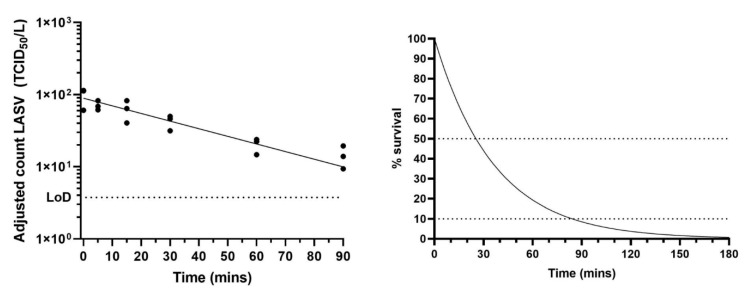
Aerosol survival of Lassa virus. (**Left**) Lassa virus was aerosolised in tissue culture media and held in a Goldberg Drum at medium relative humidity (50–60%). Impinger samples were taken at set time points and enumerated by TCID_50_ assay. Three aerosol experiments were performed: each time-point was assayed in triplicate. Counts were adjusted for dilution effect of sampling and mean adjusted counts of each of the 3 experiments plus a best fit line are shown. The limit of detection (LoD) of the TCID_50_ assays is shown as a dotted line. (**Right**) The theoretical decay of Lassa virus in TCM with decay as a percentage of starting amount shown. Horizontal lines at 50% represent half-life and at 10% give an indication of time for starting amount to drop by 90% (1× Log_10_).

The biological decay rate of Lassa virus within small particle aerosols was measured using the rotating Goldberg drum. Drum experiments were performed in triplicate (at 50–60% relative humidity and 22 °C ± 3 °C). The biological decay rate of Lassa virus was defined by the decay constant, K, and determined to be −0.02726; this is equivalent to a biological decay rate of 2.69 % min^-1^. Viable titres of Lassa virus could still be quantified after 90 min in a dynamic aerosol (Figure 2). Under such conditions, the half-life of Lassa virus in a small particle aerosol was estimated at 25 min. The hypothetical decay, as a percentage of starting amount, can be determined (Figure 2) and indicates that under these defined experimental conditions of temperature, relative humidity and held in the dark, the starting titre of virus would have dropped by 90% within 90 min, and 99% within 3 h (Figure 2).

## 3. Discussion

In small volumes of blood and tissue culture media, samples representative of common hospital and laboratory matrices, Lassa virus did not survive for long periods of time at an elevated temperature or when exposed to the open environment (tube open, allowing the liquid to dry). Compared to other Hazard Group 4 viruses studied under similar conditions, Lassa virus was found to be less stable than Nipah virus, which could be detected after three days under identical conditions [9]. When dried onto surfaces in tissue culture media or guinea pig serum, neither Ebola virus nor Marburg virus could be recovered from any substrate stored at room temperature [10]: this suggests that Lassa virus may be more stable than these two filoviruses as viral recovery in both blood and TCM was achieved, from open tubes, at 24 h. Inactivation kinetics of Lassa virus dried onto solid surfaces also indicated increased stability of Lassa virus compared to Ebola virus [11]. However, when filoviruses were maintained in liquid (similar to the sealed conditions tested here), they were found to be stable over a long period [10]. As a result of the increased decay rate under most conditions, prolonged time periods were not included in this study; however, it would be expected, as with many other viruses that, in sealed tubes kept at room temperature, Lassa virus would persist for longer periods. Direct comparison of the current data with other published observations may not be relevant as experimental conditions and procedures can vary. The current data may broadly suggest however that Lassa virus is less stable than Crimean Congo Haemorrhagic Fever virus, which was still detected at 11 days at 20 °C, but could not be detected after 7 h at 37 °C in cell culture media in wet conditions on a metal surface [12]. When dried, CCHF virus could not be detected after 24 h and this would be more comparable to our ‘open tube’ conditions where droplets dried. Of the 24 ‘open tube’ samples tested, no viable virus (22 samples) or extremely low levels of viable virus at the limit of detection (two samples) could be detected in any replicates at 48 h. 

High levels of viremia have been reported in non-human primates infected with Lassa virus [13,14]. In marmoset blood >5–7 log_10_ PFU/mL was reported [13] and >10^6^ PFU/mL was reported in the blood of crab-eating macaques [14]. In humans, titres in the blood greater than 10^3.6^ TCID_50_/mL are associated with a case fatality rate of over 70% [15]. The titre of the virus stock used in these experiments and the need to dilute into blood to create a representative substrate meant our starting titres were not as high as what has been reported in infected animals. However, we have determined decay rates so the data can be extrapolated and would be expected to show a similar pattern irrespective of starting titre, i.e., viable virus will be stable at room temperature if in a liquid format but will decay rapidly at elevated temperatures and/or if desiccated. Blood represents a complex matrix where the virus may be protected from damage that occurs during drying processes. Blood is also the basis for several Lassa virus diagnostic tests [16,17] and therefore was deemed to be a relevant and common sample type with which to investigate viral survival. The two temperatures selected for investigation represented indoor conditions (air conditioned laboratory) and an elevated temperature that represented the higher temperatures that might occur in West Africa [18]. Inclusion of a third sample type of rodent urine, if available, would be useful for inclusion in future survival studies to understand more about the risk of natural transmission. Small volumes were used to represent droplets that might form during, or remain after, laboratory or medical procedures that might be missed during disinfection and could persist and pose a hazard to others. Depending on the conditions, viable virus could remain for several days. Larger volumes, such as would occur in a spill, would behave differently due to the surface area and evaporation dynamics, and we anticipate that larger volumes would be observed and therefore managed with disinfection. 

In an experimentally generated small particle aerosol held in dark conditions at 20 °C and 50–60% relative humidity, viable Lassa virus could still be detected after an hour. The stability of Lassa virus (Josiah strain) in an aerosol has been reported with a biological decay rate of 3.2% per minute and biological half-life of 21 min at 24 °C and 55% relative humidity [19]. This was comparable to that reported here, where a biological decay rate of 2.7% and a biological half-life of 25 min was calculated. The experimental conditions and viral strains varied between the studies, however the comparable results support the data reported here. Stephenson et al. found that at 24 °C decay was quicker at higher relative humidity values, but at an elevated temperature of 32 °C the virus maintained viability for longer as the relative humidity increased ([19], also reviewed in [20]). In general, small particle aerosols are relevant to study as they may be generated during routine laboratory and medical procedures and can also be produced by activities such as breathing and talking [21,22]. Decay data can be used in modelling to determine how long viable virus will remain after accidental or deliberate aerosol generation. Many factors will influence aerosol decay including, but not limited to, temperature, relative humidity, the amount and type of light (UV from sunlight will have a major impact), air flow dynamics, and with indoor environments, ventilation and filtration.

The decay rate of Lassa virus in a small particle aerosol was similar to that of Influenza virus with a half-life of 32 min [23]. Lassa virus appeared more stable than the filoviruses where the half-life of Ebola virus was determined as 15 min and 14 min for Marburg virus [10]. Lassa virus is less stable in an aerosol than estimated for the Smallpox virus (determined using Vaccinia virus), and several encephalitic viruses including Venezuelan equine encephalitis virus, Japanese encephalitis virus, and St. Louis encephalitis virus [20]. Lassa virus was also less stable than several important Coronaviruses [24,25]. 

Many factors including environmental conditions and the phenotype of the virus (for example whether it is enveloped) as well as physical properties of surfaces contribute towards the survival of viruses on various surfaces (reviewed in [26]) and in aerosols. It is therefore difficult to extrapolate data from one virus type to another; however, it remains important to have a fundamental understanding of the survival properties of emerging viruses to help inform risk assessments, determine decontamination and transmission rates, and inform mitigation strategies in the event of an outbreak.

## 4. Materials and Methods 

### 4.1. Virus Growth and Enumeration

Lassa virus GA391 from a case in Nigeria [27] was kindly supplied by Public Health England (UK) and grown in Vero C1008 cells (ECCAC Cat. No. 85020206) maintained in tissue culture media (TCM), Dulbecco’s minimum essential media (DMEM, Gibco, Thermo Fisher, Loughborough, UK) supplemented with 2% Foetal Calf Serum, 1% L-glutamine and 1% Penicillin/streptomycin (all Sigma, Gillingham, UK). Lassa virus was harvested after five days growth in cells at 37 °C/5% CO_2_ and was enumerated using the endpoint fifty percent tissue culture infectious dose (TCID_50_) assay. Briefly, Lassa virus was ten-fold serially diluted in 96 well plates previously seeded with Vero C1008 cells. After 5–7 days of incubation at 37 °C/5% CO_2_, all wells were observed under the microscope and scored for presence or absence of cytopathic effects. The 50% end-point was then calculated using the method of Reed and Muench [28]. All work with Lassa virus was performed under ACDP Containment Level 4/ BSL-4 conditions. The stock used in all studies was at 2 × 10^6^ TCID_50_/mL. 

### 4.2. Liquid Survival Studies

Studies were similar to those described previously with Nipah virus [9]. Briefly, 20 µL aliquots of Lassa virus stock in DMEM TCM (Gibco), or 20 µL aliquots of a 1:10 dilution of Lassa virus stock in purchased Sprague Dawley adult rat blood (Charles River, Margate, UK) were placed in 2 mL Sarstedt Screw Cap Micro tubes. Tubes were stored at room temperature (19–22 °C, 50–70% relative humidity) within a microbiological safety cabinet (MSC) held at negative pressure (approximately −300 Pascals with 200−300 air changes an hour) or held in a hot block (Stuart Scientific) held at 30 °C at the same relative humidity and within the same microbiological safety cabinet. Tubes were stored sealed (lids on) or open (lids off). At daily intervals, three aliquots from each condition were removed, 1 mL TCM was added to each tube irrespective of how much original liquid remained, and the sample vortexed for 10 s prior to a TCID_50_ assay being performed on the resuspended droplet. The experiment was performed on two separate occasions from the same initial starting stocks. Analysis was performed in GraphPad Prism v8. To determine the biological decay rate, a line of best fit was added using the one phase decay equation: Y = (Y_0_ − Plateau) * e^−KX^ + Plateau, where Y_0_ is the Y value when X (time) is zero, Plateau is the Y value at infinite times and K is the rate constant. 

### 4.3. Viral Survival Studies Within Aerosols Using the Rotating GOLDBERG Drum

Harvested cell culture supernatant of Lassa virus was aerosolised using a Collison nebuliser and AeroMP system (BiAera Hagerstown, MD, USA) to produce small particle aerosols of 1–3 µm diameter. A rotating Goldberg drum methodology was similar to that described previously [10,21]. Briefly, virus suspension was sprayed for 5 min into a 40 L rotating Goldberg drum and then allowed to mix for 2 min. The aerosol was maintained at 50–60% relative humidity and 22 °C ± 3 °C. Impinger samples (at a rate of 4 L/min) were taken for 1 min at pre-determined intervals into 3 mL TCM in a midget impinger (SKC Ltd., Blandford Forum, UK). Impinger samples were assayed by TCID_50_ assay. Data were corrected for the dilution effect of make-up air per time-point. To determine the biological decay rate, a line of best fit was added using the exponential decay equation (Y = Y_0_*e^kX^) where Y0 is the Y value when X (time) is zero and K is the rate constant. 

## Figures and Tables

**Table 1:** Decay constant, K, for Lassa virus under different conditions (Hour^−1^).

Substrate	Temperature	Open	Sealed
**TCM**	19–22 °C	0.250	0.149
	30 °C	0.411	0.325
**Blood**	19–22 °C	0.194	ND
	30 °C	ND	0.224

ND: Not determined.

## Disclaimer

© Crown copyright (2020): Dstl. This material is licensed under the terms of the Open Government Licence except where otherwise stated. To view this licence, visit http://www.nationalarchives.gov.uk/doc/open-government-licence/version/3 or write to the Information Policy Team, The National Archives, Kew, London TW9 4DU, or email: psi@nationalarchives.gov.uk.
